# Laparoscopic repair for vesicouterine fistulae

**DOI:** 10.1590/S1677-5538.IBJU.2014.0391

**Published:** 2015

**Authors:** Rafael A. Maioli, André R. S. Macedo, André R. L. Garcia, Silvio H. M. de Almeida, Marco Aurélio Freitas Rodrigues

**Affiliations:** 1 Disciplina de Urologia, Departamento de Cirurgia da Universidade Estadual de Londrina. Paraná, PR, Brasil

## Abstract

**Objective:**

The purpose of this video is to present the laparoscopic repair of a VUF in a 42-year-old woman, with gross hematuria, in the immediate postoperative phase following a cesarean delivery. The obstetric team implemented conservative management, including Foley catheter insertion, for 2 weeks. She subsequently developed intermittent hematuria and cystitis. The urology team was consulted 15 days after cesarean delivery. Cystoscopy indicated an ulcerated lesion in the bladder dome of approximately 1.0cm in size. Hysterosalpingography and a pelvic computed tomography scan indicated a fistula.

**Materials and Methods:**

Laparoscopic repair was performed 30 days after the cesarean delivery. The patient was placed in the lithotomy position while also in an extreme Trendelenburg position. Pneumoperitoneum was established using a Veress needle in the midline infra-umbilical region, and a primary 11-mm port was inserted. Another 11-mm port was inserted exactly between the left superior iliac spine and the umbilicus. Two other 5-mm ports were established under laparoscopic guidance in the iliac fossa on both sides. The omental adhesions in the pelvis were carefully released and the peritoneum between the bladder and uterus was incised via cautery. Limited cystotomy was performed, and the specific sites of the fistula and the ureteral meatus were identified; thereafter, the posterior bladder wall was adequately mobilized away from the uterus. The uterine rent was then closed using single 3/0Vicryl sutures and two-layer watertight closure of the urinary bladder was achieved by using 3/0Vicryl sutures. An omental flap was mobilized and inserted between the uterus and the urinary bladder, and was fixed using two 3/0Vicryl sutures, followed by tube drain insertion.

**Results:**

The operative time was 140 min, whereas the blood loss was 100ml. The patient was discharged 3 days after surgery, and the catheter was removed 12 days after surgery.

**Discussion:**

Laparoscopy has advantages over open surgery in that it is associated with less pain, shorter length of hospital stay, better cosmesis, quicker recovery, and equal efficacy. Although cases of VUF are rarely noted, the laparoscopic skill obtained through other urological procedures suggest, that laparoscopic repair may be the procedure of choice for such cases (2). The reported operative time for the laparoscopic repair of VUF in the literature varies between 140 and 220 min (3). However, laparoscopic techniques should be considered as a mode of abdominal access and should not influence the method of surgical repair. Surgical success should depend on the adherence to good technique rather than the approach. Hence, this method appears to be a viable alternative for surgeons experienced with laparoscopic suturing techniques.

**Conclusion:**

Laparoscopic repair appears to be a viable alternative for surgeons experienced with laparoscopic suturing techniques.

## INTRODUCTION

Vesicouterine fistulae (VUF) comprise 2-9% of all cases of urogenital fistulae. Only 5% of patients respond to conservative management, whereas other patients require definitive surgical repair (1). Although laparoscopy provides better visualization through magnification, this technique is difficult to learn. We hypothesized that the experience gained through other pelvic laparoscopic procedures, such as ureteral re-implantation, correction of vesicovaginal fistulae, and radical prostatectomy, may enhance the ability of urologists to perform this procedure with traditional laparoscopic intracorporeal suturing, without the need for any special tools.

## ARTICLE INFO

**ARTICLE INFO Available at:** **http://www.brazjurol.com.br/videos/september_october_2015/Maioli_1030_1031video.htm**

**Int Braz J urol. 2015; 41 (video #8): 1030-1**
