# Supplementary material for: Laparoscopic repair for vesicouterine fistulae
Source: Int Braz J Urol. 2015 Sep-Oct;41(5):1030–1. doi: 10.1590/S1677-5538.IBJU.2014.0391 (PMC4756983; doi:10.1590/S1677-5538.IBJU.2014.0391)
Supplement: Related texts

# Letter: EDITORIAL COMMENT

**Authors:** Hubert S Swana

**Affiliations:** Nemours Children's Hospital, Orlando, 13535 Nemours Parkway, Orlando, Florida, 32827, USA, Fax: + 1 407 650-7266. E-mail: hswana@nemours.org

This video nicely depicts the laparoscopic repair of a vesicouterine fistula. Vesicouterine fistulae are uncommon and have been estimated to account for only 1% to 4% of all genitourinary fistulae. (1) Conservative measures are usually attempted first. They include bladder decompression with an indwelling catheter and medical management to induce amenorrhea. (2) In select patients cystoscopic fulguration of the vesicular portion of the fistula has also been proven to be effective. (3) Laparoscopic and robotic repairs have been reported as alternatives to open surgery. Regardless of technique, adherence to the principles for genitourinary fistula repair will maximize the chances for success. Wide exposure and excision of scar tissue around the fistula; tension-free closure of the wound in multiple layers; and utilization of an omental or myouterine flap are all possible with minimally invasive techniques. (4, 5)

## References

[1] Iloabachie GC, Njoku O (198). Vesico-uterine fistula. Br J Urol.

[2] Jóźwik M, Jóźwik M (1999). Spontaneous closure of vesicouterine fistula. Account for effective hormonal treatment. Urol Int.

[3] Molina LR, Lynne CM, Politano VA (1989). Treatment of vesicouterine fistula by fulguration. J Urol.

[4] Das Mahapatra P, Bhattacharyya P (2007). Laparoscopic intraperitoneal repair of high-up urinary bladder fistula: a review of 12 cases. Int Urogynecol J Pelvic Floor Dysfunct.

[5] Chang-Jackson SC, Acholonu UC, Nezhat FR (2011). Robotic-assisted laparoscopic repair of a vesicouterine fistula. JSLS.
